# Efficiency, Usability, and Outcomes of Proctored Next-Level Exams for Proficiency Testing in Primary Care Education: Observational Study

**DOI:** 10.2196/23834

**Published:** 2021-08-16

**Authors:** Birgitte Schoenmakers, Johan Wens

**Affiliations:** 1 Department of Public Health and Primary Care KU Leuven Leuven Belgium; 2 Department of Primary and Interdisciplinary Care University of Antwerp Antwerpen Belgium

**Keywords:** primary care, education, graduate, medical education, testing, assessment, app, COVID-19, efficiency, accuracy

## Abstract

**Background:**

The COVID-19 pandemic has affected education and assessment programs and has resulted in complex planning. Therefore, we organized the proficiency test for admission to the Family Medicine program as a proctored exam. To prevent fraud, we developed a web-based supervisor app for tracking and tracing candidates’ behaviors.

**Objective:**

We aimed to assess the efficiency and usability of the proctored exam procedure and to analyze the procedure’s impact on exam scores.

**Methods:**

The application operated on the following three levels to register events: the recording of actions, analyses of behavior, and live supervision. Each suspicious event was given a score. To assess efficiency, we logged the technical issues and the interventions. To test usability, we counted the number of suspicious students and behaviors. To analyze the impact that the supervisor app had on students’ exam outcomes, we compared the scores of the proctored group and those of the on-campus group. Candidates were free to register for off-campus participation or on-campus participation.

**Results:**

Of the 593 candidates who subscribed to the exam, 472 (79.6%) used the supervisor app and 121 (20.4%) were on campus. The test results of both groups were comparable. We registered 15 technical issues that occurred off campus. Further, 2 candidates experienced a negative impact on their exams due to technical issues. The application detected 22 candidates with a suspicion rating of >1. Suspicion ratings mainly increased due to background noise. All events occurred without fraudulent intent.

**Conclusions:**

This pilot observational study demonstrated that a supervisor app that records and registers behavior was able to detect suspicious events without having an impact on exams. Background noise was the most critical event. There was no fraud detected. A supervisor app that registers and records behavior to prevent fraud during exams was efficient and did not affect exam outcomes. In future research, a controlled study design should be used to compare the cost-benefit balance between the complex interventions of the supervisor app and candidates’ awareness of being monitored via a safe browser plug-in for exams.

## Introduction

The COVID-19 pandemic has heavily affected education and assessment programs. Flemish (Belgian) universities were, as those in many other countries, not sufficiently prepared to fluently switch from analogous teaching and testing to digital teaching and testing. Moreover, the COVID-19 measures have also necessitated physical distancing before and during exams, which has resulted in a lower number of candidates participating in exam sessions. This situation has resulted in complex planning for managing human resources, locations, and adequate equipment.

The challenge of reorganization is even more impressive in interuniversity collaborations for education. The Advanced Master of Family Medicine program in Flanders, Belgium, is formally organized and is offered by 4 Flemish universities. This collaboration comprises a common administration, common courses and examinations, common residencies, and separate registers of residents for the university of their choice. In the three phases of the advanced master program, we have over 900 residents in training and in education. The planning of examinations is therefore a complex logistic and administrative matter. To address this logistic challenge, we built (more than 1 decade ago) an intelligent, comprehensive, and interactive assessment platform. From other authors, we have learned that (medical) students are in favor of computerized exams, and these exams may enhance learning experiences and effects without affecting final test outcomes [1-4]. The platform we developed provides an interface for summative and formative knowledge testing (in 6 questions formats) and Objective Structured Clinical Examination–performance and proficiency testing.

In general, it is not desirable to postpone or reschedule high-stakes tests or assessments [5,6]. Such exams are often organized apart from the regular exam schedule. The exam regulations and planning process of the proficiency test for admission to the Advanced Master of Family Medicine program in Flanders are also different from those of regular exams. Further, the same proficiency test is organized for the four Flemish universities. The test involves a 3-stage procedure that starts with an administrative stage. This is followed by the actual exam, and the test is finalized with a jury exam for candidates who failed the exam in stage 2. The actual exam is a machine-assisted test that is conducted on the digital assessment platform. This whole procedure has been conducted since 2016, and this format has proven to be reliable, acceptable, and feasible [7].

The combination of planning an off-schedule exam and offering exams on campus (ie, the ruling policy) while adhering to the original exam format has forced us to opt for a creative solution. We decided to prioritize the format of this high-stakes test and organize—in addition to the original on-campus exam—a proctored off-campus exam. Web-based exams (or remote exams) for high-stakes assessments are considered user-friendly and cost-friendly and are flexible in terms of planning, but their validity is questionable if fraud cannot be ruled out [3,8-10]. The development and administration processes of proctored exams are still under construction and have been boosted during the COVID-19 pandemic [5]. The most commonly applied antifraud measures are direct face visualization and visual verification through a webcam [11-13]. However, screening all of the webcam images of large groups of students is time consuming, and image quality is highly dependent on webcam technology, a stable internet connection, and background lighting [11,12].

To minimize the occurrence of fraudulent events during the exam, we developed a web-based supervisor app for tracking and tracing candidates’ behaviors during the exam. The technology we have built and applied goes beyond that of traditional proctored exam systems that focus on tracing sounds and images. In addition to real-time supervision (image and sound), all student behavior is recorded and logged for posttest reassessment in case of suspiciousness. In this paper, we report on the efficiency and usability of our antifraud measures and the impact that a proctored exam has on exam outcomes.

## Methods

### Study Design

Through collaboration with the developers of the assessment platform and through discussions with the coordinators and exam supervisors (called the expert group) of the Advanced Master Education program, we determined the criteria and conditions for designing the supervisor app. The application operated on the following three levels: the recording of actions, analyses of behavior, and live supervision. First, during the exam, the system recorded data from three sources—the computer screen, the camera, and the microphone. These recordings were immediately encrypted and saved on a secured server. Second, the supervisor app used a pattern recognition algorithm for response, clicking behavior, and time stamp analyses (Textbox 1). The application also analyzed individual behavior as well as correlations across (collaborating) candidates. Each event was given a score. Every suspicious event increased the final suspicion rating by 0.1. Based on the consensus of the expert group, a candidate with a suspicion rating of 0.5 or higher was considered suspicious. The tracking data were stored in the users’ browsers and sent to the server after 20 nonsuspicious events occurred or when suspicious events occurred. If the suspicion rating rose above 0.5, the exam submission was flagged, and a report on the flagged behavior was downloaded for assessment. Suspicious events were defined as switching to another browser, returning to the exam page, closing the exam page, disconnecting from the internet, and making sounds and noises.

**Types of events that were tracked and traced by the supervisor app.**

**Events**

- Blur page (suspicious)
  This means that the user switched to another browser tab or another program.
- Focus page (suspicious)
  This means that the user returned their focus to the page.
  Focus page events can be used (after blur page events) to determine how long the user had been gone.
- Close page (suspicious)
  This means that the user closed the current tab or opened a different page in the same tab.
- Load page
  This event occurs when the user loads a page.
  Load page events can be used (after close page events) to determine how long the user had been gone.
- Keyboard events
  Logged with a basic key logger
- Connected to the internet 
- Disconnected from the internet (suspicious)
  The user can stop their internet connection to stop video recordings. This data were sent to the server when the user reconnected to the internet.
- Mouse clicks
  Includes click location coordinates
- Sound detected (suspicious)
  The app tracks any noise above certain volume levels with a duration of >1 ms.
- Administrator started watching
  This is marked in blue on the tracking page.
- Administrator started talking
  This is marked in blue on the tracking page.
- Finalization of the test component
  This is needed because when a user completes the test component, a close page event is triggered.

The third level of supervision allowed for optional human oversight during the examination. The human monitor could immediately join the live feed of each candidate to obtain more information or to send a warning to candidates in private. In case the supervisor app crashed, the affected candidates were able to switch to the Safe Exam Browser (Eidgenössische Technische Hochschule Zürich), which allows only the exam interface to remain accessible on a user’s computer.

In addition to the technical solutions, we expected the application to have a preventive effect on candidates’ behaviors. Candidates were comprehensively briefed in advance on how to install the application, on how to test it, and on the specific features of the supervising technology. We used an animation video and a written instruction form for the briefing.

A voluntary panel that consisted of experienced teachers and exam supervisors tested the supervisor app in 2 sessions. These test participants were asked to behave in a suspicious manner (ie, talking, making noise, turning away from the screen, using the internet, typing, clicking, and using the mouse). After the first session, we made the following adjustments to the application and the procedure: we isolated the suspicious sound events from the other suspicious events, and we increased the overall suspicion rating from 0.5 to 1.

The comparison group was composed of the on-campus candidates who were supervised under the usual circumstances and conditions. To assess efficiency, we logged technical issues and the interventions that were used during the exam. Technical issues were defined as those reported by students, and interventions referred to the active responses of the team. To analyze the impact that the supervisor app had on students’ exam outcomes, we compared the scores of the proctored group to those of the on-campus group. To test usability, we counted the number of suspicious students and behaviors.

All students who applied for admission to the advanced master program were included in this study. To take the exam, candidates were free to register for off-campus participation or on-campus participation. On campus (n=4), a human supervisor was present, and candidates used the campus’ gear. For off-campus monitoring, we had an experienced supervising team of 6 staff members. A team of 2 developers was also fully available during the exam. The off-campus supervisors were able to send notifications or warnings to candidates who were behaving suspiciously, and these supervisors intervened in cases involving technical issues.

The actual exam and the associated procedures were set up in the same manner as those that were conducted before the COVID-19 pandemic; all candidates completed the same exams, and candidates who failed the machine-assisted test (the actual exam) were invited to a jury exam after 1 week. Candidates who were flagged as those exhibiting suspicious behaviors during the exam or in postexam analyses were also invited to the jury exam.

### Ethical Approval

Ethical approval was not required for this study. Consent for the administration of the proctored exam was obtained from the Permanent Education Commission of the Faculty of Medicine of KU Leuven.

### Data Set

The complete data set is available upon simple request and can be sent as a link to a Google Drive directory.

## Results

A total of 593 candidates subscribed to the exam. Of these candidates, 472 (79.6%) used the supervisor app for off-campus exams and 121 (20.4%) were present on campus (Table 1). The test results of both the off-campus and on-campus groups did not differ significantly (*P*=.15).

**Table 1:** Participation and test results of off-campus students and on-campus students.

Characteristics		Off-campus students	On-campus students	*P* value^a^
Candidates (N=593), n (%)		472 (79.6)	121 (20.4)	N/A^b^
**Candidates at each university, n (%)**				
	Leuven University	227 (84.1)	43 (15.9)	N/A
	Antwerp University	29 (35.8)	52 (64.2)	N/A
	Brussels University	13 (50)	13 (50)	N/A
	Gent University	203 (94)	13 (6)	N/A
Average test results (out of 100)		72	72.8	.15

^a^The *P* value was determined via a one-tailed pooled *t* test.

^b^N/A: not applicable.

Overall, we registered and solved 15 technical issues that occurred during the off-campus exam (Table 2). Of these issues, 8 were the result of software problems (in particular, loading a reading text in a new tab). Further, 2 candidates experienced a negative impact on their exam performance due to technical issues. The development team made one of these candidates switch to the Safe Exam Browser to complete the exam. Further, based on the postexam analyses and after deliberation, the coordinator exempted the other candidate of the jury exam.

**Table 2:** Comparison of exam procedures and outcomes of the off-campus exam and on-campus exam.

Characteristics		Off-campus exam	On-campus exam
Technical issues, n		15	1
Technical issues with an impact on the exam, n		2	0
**Type of issue, n**			
	Internet failure	2	0
	Hardware issue	4	1
	Camera crash	1	0
	Software issue	8	0
Average suspicion score		0.4	N/A^a^
Median suspicion score		0.3	N/A
**Suspicious candidates, n (%)**			
	Detected by the application	22 (4)	N/A
	Flagged by supervisors	2 (<1)	N/A
	Flagged due to noncritical events (≤1 event)	455 (96)	N/A
	Flagged without events	15 (3)	N/A
	Flagged due to noise events	472 (100)	N/A
**Interventions during the exam, n**		8	1
	Technical interventions	2^b^	0
	Warnings to candidates	1^c^	0

^a^N/A: not applicable.

^b^Technical interventions were needed for 2 individuals.

^c^Warnings were given to 1 group due to background noise.

Of the 593 candidates, the application detected 22 (3.7%) with a suspicion rating of >1. All cases were the result of 1 or more noise events (background noise). These students received an immediate but nonoffensive request to stop all background noise without further consequences or impacts on the progression of their exams. Additionally, the supervisor sent a request to all students to reduce background noise. All other noncritical events included leaving the webpage, closing a page, or typing text. Live monitoring and a postexam review of the recordings confirmed that all of these events occurred unintentionally and without fraudulent intent. In the on-campus group, we did not detect suspicious or fraudulent students.

The monitoring supervisors flagged two candidates who were typing more than the expected amount (in a multiple-choice exam). After the review of the recordings, it was found that these two candidates were using the “control find” function to search for words in the reading text. During the exam, the supervisors intervened 8 times due to technical issues, warned 2 candidates to stop talking to themselves, and sent a group message to ask candidates to reduce background noise.

## Discussion

This pilot observational study demonstrated that a supervisor app that records and registers behavior was accurate because it was able to detect all suspicious events without having an impact on exam performance. The efficiency of the proctored supervisor system seemed low, as shown by the availability of a 12-person bystander team, which monitored and solved a relatively low number of technical issues.

Students have shown certain preferences for different exam formats depending on how much confidence they have in their techniques, the organization, exam procedures, and exam outcomes and how confident they are with technological aspects and issues [9,12,14,15]. The unbalanced number of registrations for off-campus and on-campus exams among the universities was striking, but this can be explained by the availability of infrastructure (classes and information technology gear). We indeed found that the candidates from larger universities were more likely to register for an off-campus exam. The psychometrics of the exam were comparable between the off-campus and on-campus groups. Therefore, we can conclude that there was probably no bias induced by the voluntary option for participating in off-campus exams or on-campus exams. Other authors have compared the scores of proctored exams to scores of traditional exams that were conducted in the past several years and found no meaningful differences [6,8,14].

The major weakness of proctored exam systems lies in the occurrence of technical or technological failures and in a lack of user experience [2,4,12,16]. The number of technical issues was low and was mainly related to software issues (technology issues). The complexity of the application (the registration of behavior, recording, etc), in combination with a multicomponent exam with different questions types, necessitated very performant technological equipment. In one case, a technical issue led to the interruption of the exam of the affected candidate. The supervisors and the development team solved all other issues within an acceptable time span. However, these issues revealed that the system was vulnerable to the low quality of the personal gear of the candidates. Therefore, education institutes should guarantee a fair and safe exam environment for every candidate by offering high-quality infrastructure and logistics [4,12,13].

During the entire exam, a team of 12 persons was constantly monitoring and maintaining contact with the candidates. Although we did not perform a cost-benefit analysis, we assume that the whole procedure does not save costs, since the on-campus exam opportunity also remained available [4].

On average, candidates induced 3 events, of which the noise and sound event was the most prevalent and critical. The development team increased the threshold sound level during the exam, since the application immediately (in the first few minutes) flagged all candidates. Sound is indeed difficult to avoid and control (we noticed children crying, birds singing, candidates talking out loud, doors being slammed, street work being conducted, etc) [11,12].

A very small number of candidates was flagged as suspicious by the application, but due to the live monitoring of the exam, they were exempted from suspicion. The supervisors reviewed the recordings of two candidates who were flagged by the system and via the live monitoring of the exam. The records did not reveal any fraudulent behavior. During the exam, the team was constantly watching the students, and the system continuously logged behaviors. Additionally, students were very aware of the presence of the supervisors. They were also aware of the fact that they were being watched and screened. Further, the exam was performed within a restricted time span; therefore, students did not have time to cheat. Consequently, students were probably very reluctant to cheat, and this might have increased their stress levels [10,13].

Prior to the exam, we thoroughly instructed the candidates on the registration of suspicious events. This single intervention might have been decisive in stopping candidates from committing fraud during this high-stakes exam [2,4,10]. A simple recording of sounds and images combined with a safe web browser that blocked all other webpages might have been just as efficient as the supervisor app [2,4]. A less comprehensive registration and recording process for suspicious events might have also reduced the number of technical issues and allowed the supervisors to focus on the live monitoring of the exam [8].

During the exams, supervisors limited the number of interventions and warnings to avoid distracting the candidates. Technical issues were solved, and supervisors sent a nonoffensive warning to only two candidates who were talking to themselves. After the exam, we only received reports of technical issues; we received no complaints regarding supervisors’ interventions or interruptions caused by the supervisors.

A weakness of the intervention is that we did not question the candidates about their experiences. This group of graduation candidates was, at the time of writing this report, very hard to reach. However, the staff and the formal faculty administration did not receive comments or objections from students in the period following the exam. Other reports have shown a relatively high satisfaction rate among master program candidates, and complaints have typically been about technical issues [16]. A second weakness is the arbitrary, expert-based threshold for suspicious behavior, which was set to 0.5. However, during the exam, it appeared that this threshold was set too low, since most students immediately obtained high scores mainly due to background noise.

A major strength of this intervention is the fact that we were able to use the application for a large group of candidates taking a high-stakes exam [2]. Additionally, we compared the exam outcomes of the intervention group with those of a control group, which was composed of candidates who preferred to take the exam on campus. We could not completely rule out bias, but since exam outcomes were comparable between both groups, we believe that the risk of bias was low. Based on other authors’ papers, we also knew that candidates’ preferences for computerized exams or paper-based exams do not influence exam outcomes [15].

### Conclusion

A sophisticated supervisor app that registered behaviors and recorded sounds and images to prevent fraud during exams proved to be efficient and did not affect exam outcomes. However, live monitoring and having a team on standby for solving technical issues resulted in a high amount human workload. In future research, a controlled study design should be used to compare the cost-benefit balance between the complex interventions of the supervisor app and candidates’ awareness of being monitored via a safe browser plug-in for exams.
